# Lkb1 Deficiency Alters Goblet and Paneth Cell Differentiation in the Small Intestine

**DOI:** 10.1371/journal.pone.0004264

**Published:** 2009-01-23

**Authors:** Boris Y. Shorning, Joanna Zabkiewicz, Afshan McCarthy, Helen B. Pearson, Douglas J. Winton, Owen J. Sansom, Alan Ashworth, Alan R. Clarke

**Affiliations:** 1 Cardiff School of Biosciences, Cardiff University, Cardiff, United Kingdom; 2 Department of Haematology, School of Medicine, Cardiff University, Cardiff, United Kingdom; 3 The Breakthrough Breast Cancer Research Centre, The Institute of Cancer Research, London, United Kingdom; 4 Peter MacCallum Cancer Institute, Research division, Cell Cycle and Cancer Genetics Laboratory, East Melbourne, Victoria, Australia; 5 The Beatson Institute for Cancer Research, Garscube Estate, Glasgow, United Kingdom; 6 Department of Oncology, Cancer Research UK, Cambridge, United Kingdom; University of Giessen Lung Center, Germany

## Abstract

The Lkb1 tumour suppressor is a multitasking kinase participating in a range of physiological processes. We have determined the impact of Lkb1 deficiency on intestinal homeostasis, particularly focussing on secretory cell differentiation and development since we observe strong expression of Lkb1 in normal small intestine Paneth and goblet cells. We crossed mice bearing an *Lkb1* allele flanked with LoxP sites with those carrying a Cyp1a1-specific inducible Cre recombinase. Lkb1 was efficiently deleted from the epithelial cells of the mouse intestine after intraperitoneal injection of the inducing agent β-naphthoflavone. Bi-allelic loss of Lkb1 led to the perturbed development of Paneth and goblet cell lineages. These changes were characterised by the lack of Delta ligand expression in Lkb1-deficient secretory cells and a significant increase in the levels of the downstream Notch signalling effector Hes5 but not Hes1. Our data show that Lkb1 is required for the normal differentiation of secretory cell lineages within the intestine, and that Lkb1 deficiency modulates Notch signalling modulation in post-mitotic cells.

## Introduction

Lkb1 is a tumour suppressor implicated in a wide range of cellular functions including inhibition of cell proliferation [1], [2]. Human *LKB1* gene mutations are known to underlie Peutz-Jeghers syndrome (PJS) characterised by intestinal hamartoma development [3], [4], [5]. It was recently shown that mesenchyme-specific Lkb1 deletion results in gastrointestinal polyps indistinguishable from those in PJS, suggesting a non-epithelial origin for intestinal hamartomas [6]. Functionally, Lkb1 phosphorylates a conserved threonine in the activation loops of AMPK and a range of AMPK-related kinases, including MARK kinases [7], [8]. The Lkb1/AMPK system has been proposed to act as an energy sensor to low ATP levels inhibiting mTOR-mediated cell growth [9]. Lkb1-mediated phosphorylation of AMPK has also been shown to be essential for the coordination between epithelial polarity and cellular energy status, suggesting one potential mechanism underlying Lkb1 tumor suppressor function [10].

To define the role played by Lkb1 in normal intestinal epithelium, we crossed mice bearing a LoxP flanked *Lkb1* cDNA cassette [11] with mice carrying a Cyp1a1-specific inducible *Cre* recombinase construct (*AhCre*). In the absence of induction, the *AhCre* transgene mediates recombination within a proportion of prostatic epithelial cells, ultimately leading to the development of prostatic intraepithelial neoplasia (PIN) by 200 days [12]. In contrast, induction of the *AhCre* transgene using β-napthoflavone results in rapid, high penetrance conditional gene deletion in the epithelium of the murine gastrointestinal tract [13]. This approach allowed us to generate Lkb1-deficient intestinal epithelium *in vivo* and assess the consequences of *Lkb1* gene function loss independently of any delayed phenotype arising in the prostate. These studies reveal, for the first time, the functional requirement for Lkb1 in the mouse intestinal epithelium.

## Results

### Lkb1 loss in the small intestinal epithelium

We generated *AhCre^+^Lkb1^fl/fl^* mice which were transgenic for both the *AhCre* transgene [13] and were homozygous for a *LoxP* flanked *Lkb1* cDNA cassette [11]. As a control we used *AhCre^−^Lkb1^fl/fl^* mice lacking the *AhCre* transgene. Both *AhCre*-positive and *AhCre*-negative mice received a series of β-napthoflavone intraperitoneal injections. These injections induced Cre recombinase expression in the intestinal crypt of *Cre*-positive animals [13], [14]. Cre-recombinase induction in the intestinal epithelium of *AhCre^+^Lkb1^fl/fl^* mice resulted in the removal of the entire kinase domain of Lkb1 (exons 4–10 of Lkb1). The regime of four daily injections produced high levels of recombination within the intestine as evidenced by qRT-PCR analysis of *Lkb1* mRNA levels, which demonstrated a 16.5 fold reduction (P<0.05, Mann-Whitney U test) by day 4 compared to β-naphthoflavone injected control mice (*AhCre^−^Lkb1^fl/fl^*).

**Figure 1:**
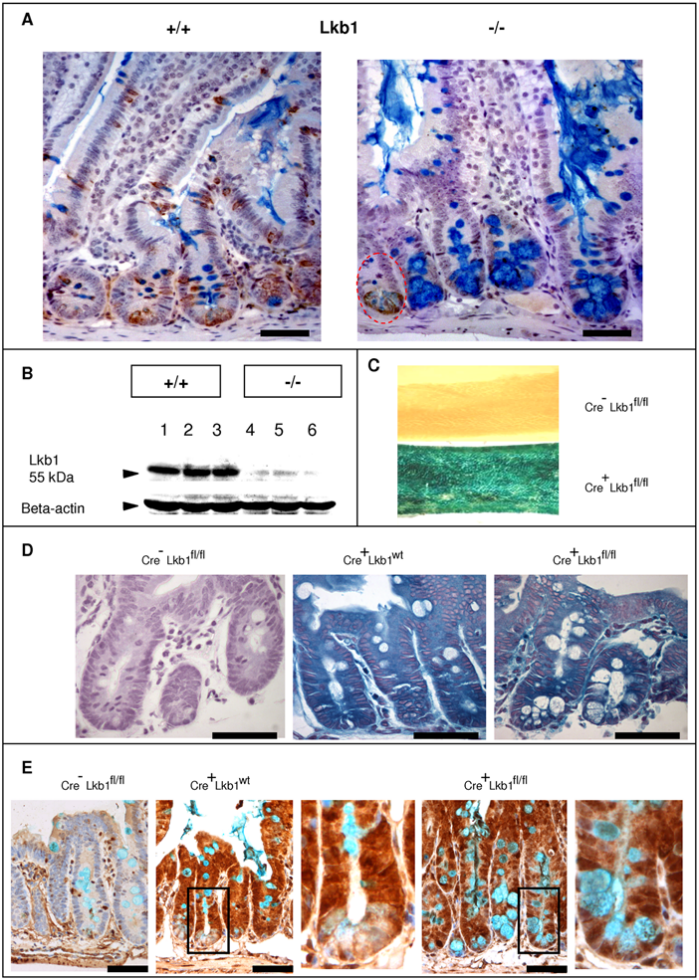
The induction of *AhCre* recombinase leads to Lkb1 loss in the epithelium of mouse small intestine. A, Immunohistochemical detection of Lkb1 expression in induced *Cre*-negative Lkb1 fl/fl (+/+) and induced *Cre*-positive Lkb1 fl/fl intestinal tissue (‘−/−’). Alcian blue staining shows the enlargement of mucin-secreting Lkb1-deficient cells. The area marked with red is the unrecombined crypt (they represent less than 5% from the whole amount of crypts) with both normal Lkb1 staining and Paneth cell morphology in induced *Cre*-positive Lkb1 fl/fl intestinal tissue (‘−/−’). B, Western blot analysis of Lkb1 expression in control (1, 2, 3) and recombined (4, 5, 6) tissue at day 6, C, D, E, Cre-mediated recombination is marked by β-galactosidase reporter gene expression. *AHCre*-positive gut shows 100% recombination after a regime of β-naphtoflavone injections compared to no recombination in *Cre*-negative gut tissue (C), Intestinal sections from Rosa26-positive mice showing the expression of β-galactosidase gene in *Cre*-positive tissue (both wt and fl/fl) and the absence of it in *Cre*-negative tissue via X-Gal staining (D) and immunostaining with anti-β-Galactosidase antibody (E). Scale bars correspond to 50 µm.

Previous studies have shown that Lkb1 mRNA is normally localised to all epithelial intestinal cells [15]. We show here that high levels of Lkb1 protein are primarily observed in the cytoplasm of goblet and Paneth cells in the small intestine (fig 1 A, “+/+”). The induction of Cre expression in *AhCre^+^Lkb1^fl/fl^* mice led to the disappearance of Lkb1 staining from Paneth and goblet cells by day 6 (fig 1 A, ‘−/−’ ) as assessed by immunohistochemistry. The level of recombination was more than 95% with very few crypts retaining Lkb1 staining (fig 1 A, ‘−/−’red circle). Western blot analysis also confirmed a drastic decrease in Lkb1 levels in the recombined tissue by day 6 (fig 1 B).

The induction of Cre recombinase by β-naphthoflavone was also monitored using the surrogate *Rosa26R* reporter locus [16]. By day 6, β-galactosidase activity was detected thoroughout the whole small intestinal tissue of *AhCre^+^Lkb1^fl/fl^* mice (fig 1, C, Cre^+^). At the same timepoint there was no detectable β-galactosidase activity in the intestines of *Cre*-negative *Lkb1^fl/fl^* mice induced with β-naphthoflavone (fig 1, C, Cre^−^). We observed a high level of recombination according to both X-Gal staining (fig 1, D) and anti-β-galactosidase immunostaining (fig 1, E) in both WT and Lkb1-deficient tissue. Paneth cells are known to have low turnover rates and would therefore be expected to be β-galactosidase negative. However, Paneth cells stained positively in Lkb1-deficient crypts suggesting an increase in the turnover of these cells in Lkb1-deficient intestines (fig 1, E).

It has been reported previously that the unrecombined mutant *Lkb1* allele is hypomorphic, with an almost 10-fold reduction in expression in some tissues [11]. Consistent with this, we observed a reduced level of Lkb1 mRNA in the intestine (P<0.05, Mann-Whitney U test), however the extent of this reduction was only 1.85 fold and was not associated with any detectable phenotypic change.

Mice harbouring the *AhCre* transgene have been reported to show spontaneous levels of recombination (in the absence of β-naphthoflavone induction) in both the kidney and genitourinary tract [14], [12], but not in the intestine [17]. Previously our group employed this spontaneous activation of *AhCre* in the genitourinary tract of male *AhCre^+^ Lkb1^fl/fl^* mice as a powerful model for prostate cancer [12]. These male mice develop PIN at approximately 200 days [12], and therefore these phenotype would not have impacted upon our intestinal studies. However, to ensure this was the case we preferentially used female mice for our experiments. We also confirmed that the spontaneous background recombination in the intestine was very low according to both β-galactosidase staining and also quantitative RT-PCR, which did not identify any differences between *Lkb1* mRNA levels in the intestines of non-induced *Cre*-negative and *Cre*-positive *Lkb1^fl/fl^* mice (data not shown)

### Lkb1 loss changes secretory cell morphology

**Figure 2:**
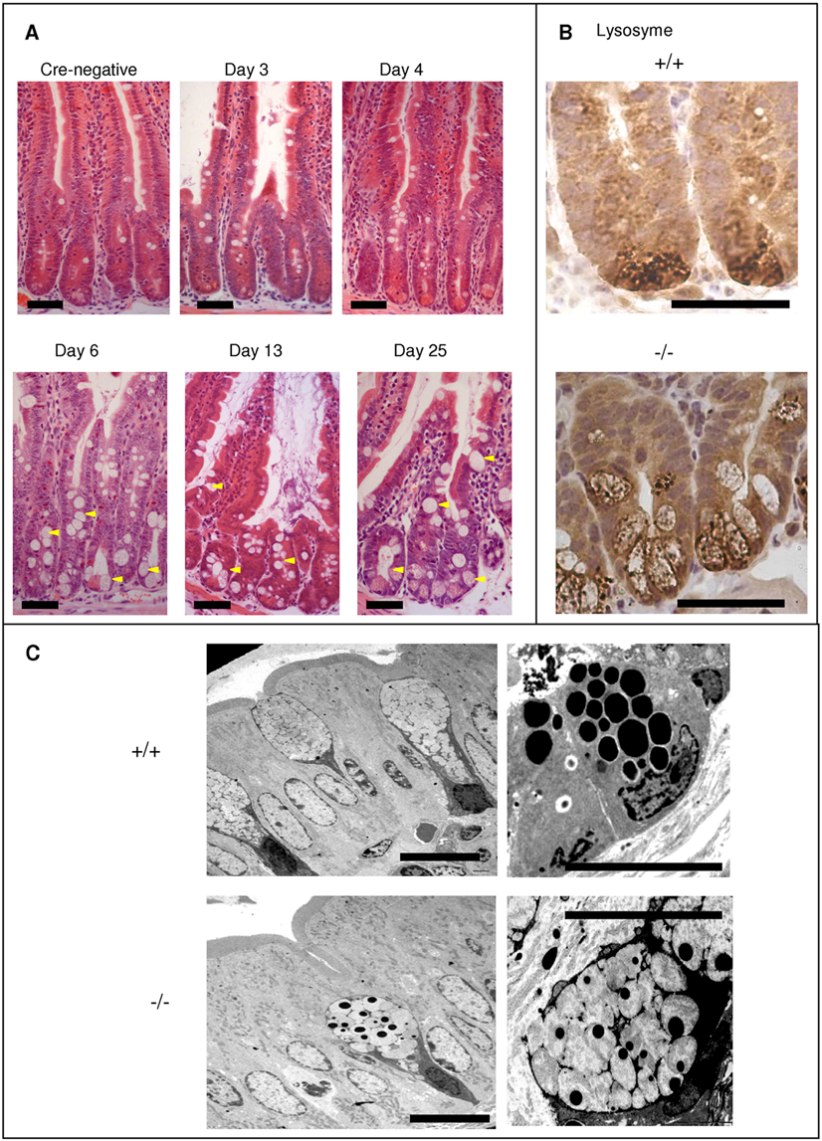
Lkb1 loss changes cell morphology in the small intestine. A, Haematoxylin and eosin staining of intestinal sections of *Cre*-negative and recombined *Cre+Lkb1fl/fl* mice at days 3, 4, 6, 13 and 25 following injection with β-naphthoflavone. Arrows show enlarged mucin-secreting cells in Lkb1-deficient epithelium. B. Lysozyme immunostaining displaying mislocalised lysozyme-secreting cells (Paneth and Intermediate cells) moving up Lkb1-deficient crypts (‘−/−’), C, Electron microscopy showing both normal goblet cells and normal Paneth cells in the intestinal tissue of wt mice (+/+) as well as an intermediate cell localised in the villus (picture on the left). Paneth cells in Lkb1-deficient crypts (‘−/−’, picture on the right) have visible changes in the bipartite structure of their secretory granules: peripheral halo composed of acid mucopolysaccharides (Spicer et al, 1967) is enlarged and central core (containing lyzosyme) is smaller in comparison with WT samples (+/+). Scale bars correspond to 50 µm (A, B) or 10 µm (C).

Lkb1 loss led to the enlargement of mucin-secreting cells, as evidenced by Alcian blue staining (fig 1, A) and also by haematoxylin and eosin staining (fig 2, A). The average size of a mucin-secreting cell at day 6 in the control tissue was 23 µm^2^ in the crypt and 33.8 µm^2^ in the villus. After the deletion of Lkb1 the figure rose to 130.7 µm^2^ in the crypt and to 94.9 µm^2^ in the villus (P<0.05, Mann-Whitney U test). *Muc2* transcript levels were elevated 5-fold in Lkb1 -deficient tissue according to quantitative RT-PCR (P<0.05, Mann-Whitney U test).

Mucin secreting cells arising in Lkb1-deficient epithelium resembled Goblet cells in shape, but they also secreted Lysozyme (fig 2, B) and contained granules normally specific to Paneth-cells (fig 2, C). Paneth cells in Lkb1-deficient crypts contained smaller Lysozyme secreting electron-dense core and larger mucin-secreting peripheral halo in comparison with WT crypts according to electron photography (fig 2, C, pictures on the right). These features are consistent with previous descriptions of Intermediate cells (intermediate between Goblet and Paneth cells), which are considered to be rare cells in transition between undifferentiated cells and more mature secretory cells [18].

### Lkb1 loss affects Hes5 expression

Goblet cell differentiation is known to be finely orchestrated by Notch signalling downstream effectors Hes1 and Hes5 at two different points and with a different outcome. Hes1 acts at the progenitor stage promoting enterocyte development and its deletion shifts the ongoing specification of epithelial cells into goblet or endocrine cell fate [19]. Hes5, on the contrary, promotes goblet cell fate but this occurs when the cells have already entered a post-mitotic stage [20].

**Figure 3:**
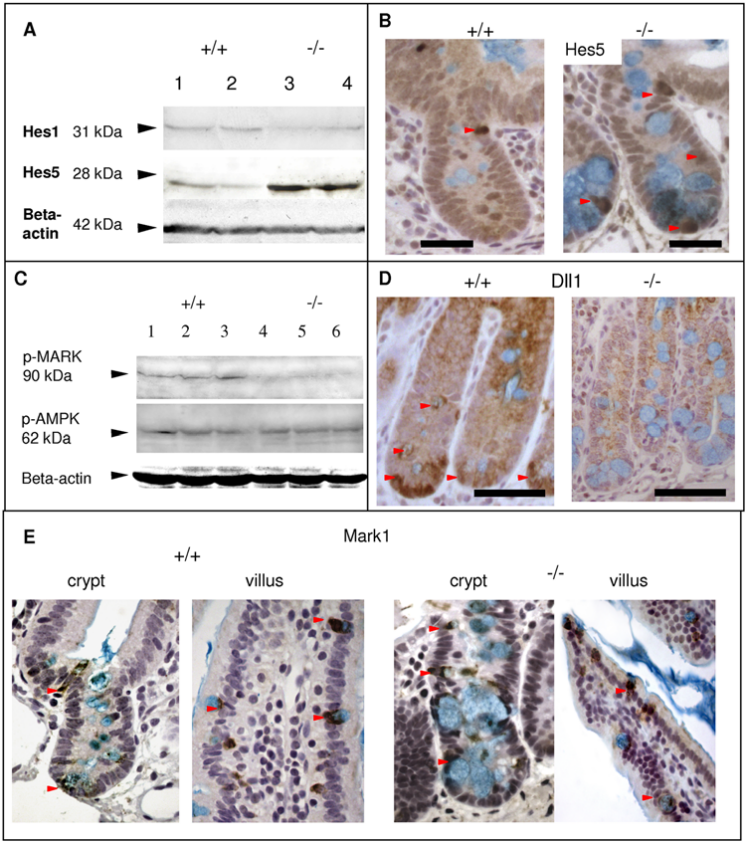
Lkb1-deficiency alters the expression of Notch signalling components and leads to the abnormal intestinal secretory cell differentiation. A, Western blot analysis of Notch signalling effectors, Hes1 and Hes5 at day 6 in induced *Cre*-negative Lkb1 fl/fl (+/+) (lanes 1, 2) and Lkb1-deficient (‘−/−’) intestinal tissue (lane 3, 4), Hes5 expression has a notable increase. B, Immunohistochemistry showing the localisation of Hes5-positive cells (pointed with red arrows) in Lkb1-deficient crypts (‘−/−’). C, Western blot analysis showing decrease in the phosphorylation of MARK kinases activation loop in Lkb1-deficient intestine (‘−/−’) while AMPK (Thr 172) phosphorylation levels are intact. D, Delta ligand (Dll1) immunostaining at day 6 in induced *Cre*-negative Lkb1 *fl/fl* (+/+) and Lkb1-deficient (‘−/−’) intestines displaying a decrease in Dll1 expression in Paneth and goblet cells of Lkb1-deficient intestines. Dll positive cells pointed with red arrows. E, Immunohistochemistry showing the localisation of Mark1 in goblet and Paneth cells both in WT (+/+) and Lkb1-deficient crypts (‘−/−’), pointed with red arrows. Scale bars correspond to 50 µm. Alcian Blue stain was performed to show mucin-secreting cells.

We therefore analysed levels of the Notch signalling effectors Hes1 and Hes5 by Western blot. Hes1 levels did not significantly change (fig 3, A), but Hes5 was notably elevated (fig 3, A). Immunohistochemical analysis of control intestines at day 6 showed Hes5-positive cells to be localised predominantly at the crypt-villus junction (fig 2, B, +/+). Lkb1-deficient samples showed that Hes5 positive cells also appear at the bottom of the crypt and amongst Paneth cells (fig 2 B, ‘−/−’).

### Lkb1 deletion in the small intestine reduces Delta ligand levels in secretory cells

**Figure 4:**
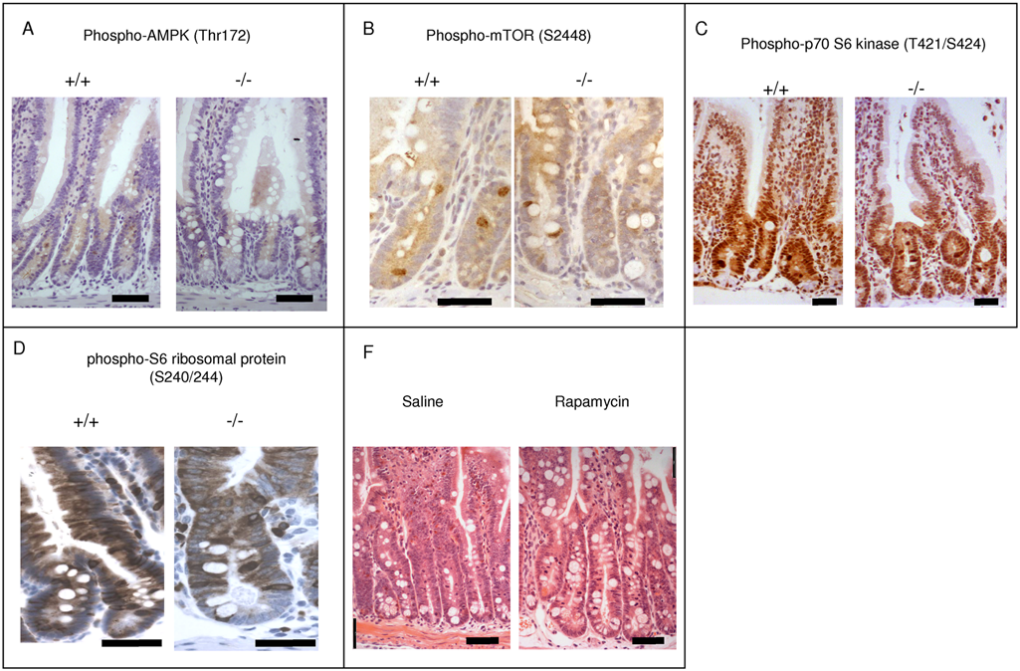
Lkb1 deletion in mouse intestinal epithelium does not affect mTOR/S6K pathway. A, B, C, D, Immunohistochemical assay showing no difference in expression of phospho-AMPK-α (Thr172) (A), phospho-mTOR (Ser-2448) (B), phospho-p70 S6 Kinase (Thr421/Ser424) (C), phospho-S6 Ribosomal Protein (Ser 240–244) (D), between induced *Cre*-negative Lkb1 *fl/fl* (+/+) and Lkb1-deficient (‘−/−’) samples at day 6. E, Haematoxylin and eosin staining of intestinal samples from Lkb1-deficient mice treated and untreated with rapamycin showing no decrease in Lkb1-deficient phenotypic features in the rapamycin-treated sample. Scale bars correspond to 50 µm.

The increase in Hes5 expression in Lkb1-negative intestinal epithelium suggested some dynamic changes in Notch signalling. Although Notch1 expression did not seem to be affected (data not shown), we observed a notable change in Delta ligand (Dll1) expression in Paneth and goblet cells. In control tissues, Dll1 was seen in virtually all cells of the crypt, with particularly high levels in Paneth and some goblet cells (fig 2 D, +/+). In Lkb1-deficient intestines Paneth and goblet cells lost Dll1 staining (fig 2 D, ‘−/−’). These changes appear to be post-translational, as quantitative RT-PCR showed no changes in *Dll1* mRNA levels. It is known that Par-1 promotes Delta ligand localization on the lateral membrane in Drosophila and the depletion of Par-1 disrupts Notch signalling [21]. Par-1 homologues in mammals are MARK kinases and they are located downstream of Lkb1 in the phosphorylation cascade [8]. We found that the phosphorylation of MARKs (using the antibody against the phosphorylated activation loop of MARK1, MARK2 and MARK3 kinases) is significantly reduced in Lkb1-deficient epithelium (fig 3, C). We examined MARK1 expression and found it to be associated primarily with goblet and Paneth cells both in the crypt and in the villus (fig 3, E). Lkb1 deletion did not change this pattern and secretory cells retained the staining (fig 3, E). Surprisingly we did not find any change in the phosphorylation of AMPK (Thr 172) by both western blot (fig 3, C) and immunostaining (fig 4, A). We also did not observe significant changes in mTOR machinery (fig 4, B, C, D) known to control cell size [22]. Furthermore, daily intraperitoneal injections of the mTOR inhibitor Rapamycin (1 mg/kg) did not reverse the phenotype observed in Lkb1-deficient intestinal epithelium (fig 4, F).

## Discussion

By creating a new mouse model with conditional gene deletion of *Lkb1*in the epithelium of the small intestine we have shown that Lkb1 is necessary for normal intestinal cell differentiation and maturation. *Lkb1* deletion not only induced an increase in the size of mucin-secreting cells, but also perturbed their morphology. Alterations in goblet cell number and elevated mucin production are common features of hamartomas characteristic of PJS [23] and specifically have been associated with loss of heterozygosity regions within hamartomas [3]. Normally, mucin is produced in goblet cells and “intermediate cells” bearing the features of both goblet and Paneth cells are rare. However, these intermediate cells were frequently observed upon *Lkb1* deletion, as indicated by both lysozyme staining (fig 2 B) and electron microscopy (fig 2 C). Secretory granules of Paneth cells from different animals tend to exhibit bipartite substructure with a peripheral halo of lower density around a large round central core of high electron density [24]. According to histochemical studies the central core of a secretory granule contained basic protein (including lysozyme) while the peripheral halo was built with acid mucopolysaccharides [25], [26]. In the case of Intermediate Paneth/goblet cells observed in Lkb1-deficient intestines the mucopolysaccharide halo is abnormally large and is stained with alcian blue (fig 1, A) whereas the lysozyme filled central core is significantly smaller than in WT (fig 2, C). This suggests that the loss of *Lkb1* creates a block in the terminal differentiation of secretory cells.

Intestinal cell specification is known to be directed by Notch signalling in mice [27], [20], Zebrafish [28] and Drosophila [29]. Following *Lkb1* deletion, we observed a decrease in Delta1 ligand (Dll1) expression in goblet and Paneth cells (fig 3, D). In zebrafish intestines Dll1 homologue is also highly expressed in secretory cells, and its inhibition leads to secretory cell expansion [28].

Notch signalling is unidirectional and it is mediated by Delta-expressing cells sending a signal and Notch-expressing cells receiving it. In Drosophila, Delta/Notch signalling involves repression of Delta in Notch-expressing cells [30]. Thus, the absence of Delta ligand in developing goblet or Paneth cells can provide a “signal receiving” role (Notch-expressing) instead of “signal sending”, which in turn can increase Hes5 expression in them.

PAR1 is known to be important for Delta ligand localisation in Drosophila [21], so we speculate that the failure in MARK (mammalian homologue of PAR1) activation in the absence of Lkb1 may directly lead to the lack of Delta ligand in Lkb1-deficient Paneth and goblet cells and subsequent deregulation of secretory cell differentiation.

The regulation of secretory cell fate by the Notch pathway is dependent on whether the cell is proliferative or post-mitotic. In proliferative cells, Notch and Hes1 repress a conversion of all dividing crypt cells into goblet cells and this conversion occurs if Notch is inhibited [27], [31]. Conversely, a very brief induction of Notch pathway drives the differentiation of post-mitotic cells into mature goblet cells via Hes5 expression [20]. In our studies, we did not observe changes in overall Hes1 levels, but Hes5 levels were notably higher, suggesting that Lkb1 deletion is more important for the differentiation at the post-mitotic stage (fig 3, A).

The failure in secretory cell terminal differentiation after Lkb1 deletion resembled the effects observed after the terminal differentiation of cell precursors into Paneth cells was blocked via SV40 T antigen expression [32]. This also led to the substitution of the mature Paneth cells with intermediate Paneth/goblet cells showing a decrease in the granule's electron-dense core diameter and expansion of the mucinous area [32]. Notch signalling misregulation observed in Lkb1-deficient intestines may also be explained by consequences arising from the altered terminal differentiation of Paneth cells development. Clearly, further studies are required to address this possibility.

Although mTOR is a known regulator of the cell size [22] and Lkb1 can suppress mTOR via AMPK phosphorylation [9] we did not find evidence of mTOR machinery misregulation in our model (fig 4) suggesting relatively intact energy sensing mechanisms.

Remarkably, given that Lkb1 has been implicated in maintenance of polarity [33], [12], we did not observe any obvious perturbations in polarity in the intestinal epithelium. It remains possible that subtle changes are occurring in the absence of Lkb1, but that these do not result in obvious changes to brush border structure and nuclear localization.

In summary, we show a complex sequence of events immediately following loss of function of Lkb1 leading to the inappropriate differentiation of secretory cells associated with abnormal expression of Notch pathway elements and consistent with many features of Notch pathway improper regulation. Our studies therefore reveal a critical role for Lkb1 in maintaining normal gut homeostasis.

## Materials and Methods

### Mice breeding and Cre recombinase induction

All experiments were carried out according to Home Office regulations. *Lkb1^fl/fl^* mice [11] were crossed to mice harbouring *Ah-Cre* transgene [13] and the offspring intercrossed to derive an outbred colony segregating for C57BL6/J, 129/Ola and C3H genomes. Mice were genotyped by PCR using DNA extracted with Puregene DNA extraction kit (Gentra systems) using following primers: *Cre*, TGACCGTACACCAAAATTTG (F) and ATTGCCCCTGTTTCACTATC (R) (991 bp product); *Rosa26R*, CTGGCGTTACCCAACTTAAT (F) and ATAACTGCCGTCACTCCAAC (R) (533 bp product); *Lkb1* allele: GTACTTCCGCCAGCTGATTGA (F) and AGTGTGACCCCAGCTGACCA (R) (320 bp and 280 bp products correspond to WT and *Lkb1^fl^* alleles respectively). Mice were intraperitoneally injected with 80 mg/kg β-naphthoflavone (dissolved in corn oil) once daily for up to 4 days to induce *Ah-Cre* gene expression and *Lkb1^fl^* allele recombination. All times indicated in the text were related to the time elapsed since the first exposure.

### β-Galactosidase analysis

7 cm long gut samples were flushed with cold water, opened out and fixed with pins on a wax plate. Following a quick fix in 2% formaldehyde/PBS/0.1% glutaraldehyde, intestines were demucified and left to stain in X-gal solution overnight (as described in [14]). The recombination was assessed by blue staining in crypts and villi and its efficiency was scored according to previous criteria [13].

### Electron Microscopy

Intestinal samples were fixed in 2% paraformaldehyde (w/v), 2% glutaraldehyde (v/v) in cold 0.1 M cacodylate buffer (pH 7.4) for 2 hours, washed briefly, and then post fixed in cacodylate-buffered 1% osmium tetroxide containing 1.5% potassium ferrocyanide for 1 hour. Specimens were washed thoroughly and then stained *en bloc* with 2% aqueous uranyl acetate for 1 hour, dehydrated through ethanol gradient and cleared in propylene oxide before, embedding in araldite resin. Semi-thin resin sections (1 µm in thickness) were cut and stained with 1% toluidine blue in borax to localise the area of interest within the tissue. Further 60 nm sections were cut and collected on formvar-coated 200 mesh copper grids and stained with 2% aqueous uranyl acid (w/v) and Reynolds lead citrate. Representative areas of gut samples were then photographed.

### RNA Extraction and qRT-PCR analysis

RNA was isolated from intestinal samples of 6–10 week old littermate mice. 2×1 cm proximal end sections of the small intestine were taken to RNAlater™ (Sigma). Tissues were homogenised in TRIZOL™ Reagent (Invitrogen) and extracted using standard Phenol-chloroform protocol. Reverse transcription was performed by using the SuperscriptII reverse transcriptase kit (Invitrogen) and random hexamers (Invitrogen) according to the manufacturer's instructions. DyNAmo SYBR green supermix (Finnzymes, GRI, Essex, U.K.) was added to appropriate cDNA samples and primers. Samples were loaded onto a white one-piece thin-wall 96-well PCR plate (Bioplastics). The PTC-200 Peltier thermal cycler (MJ Research) and Chromo4 fluorescence detector (MJ Research) were used in conjunction with Opticon Monitor analysis software (version 2.03, MJ Research) to calibrate and run the reaction. qRT-PCR was performed on all samples including reverse transcriptase negative controls. All the control samples were checked and proved to be negative on a 2% agarose gel. βeta-actin and GAPDH were used as reference genes. Primer sequences are as follows: *Beta-actin*, 5′-CTTCCTCCCTGGAGAAGAGC-3′ (forward), 5′-AAGGAAGGCTGGAAAAGAGC-3′ (reverse), *GAPDH*, 5′-CACTGAGCATCTCCCTCACA-3′ (forward), 5′-GTGGGTGCAGCGAACTTTAT-3′ (reverse), *Hes1*, 5′-TAACGCAGTGTCACCTTCCA-3′ (forward), 5′-AAGAGAGAGGTGGGCTAGGG-3′ (reverse), *Lkb1* 5′-CTCCGAGGGATGTTGGAGTA-3′ (forward), 5′-GCTTGGTGGGATAGGTACGA-3′ (reverse) *Muc2* 5′-ACATCACCTGTCCCGACTTC-3′ (forward), 5′-GAGCAAGGGACTCTGGTCTG-3′ (reverse).

All primers used were designed using primer3™ software. Reaction conditions were as follows: 95°C 30 s, 60°C 30 s, 72°C 30 s for 35 cycles. Fold change was determined as previously described using 2^−ΔΔCT^ method [34].

### Western analysis

Intestinal tissue (50–100 mg) was snap frozen in liquid nitrogen and ground using a mortar and a pestle and then solubilised in 500 µl of lysis buffer (50 mM Tris pH 7.5, 100 mM NaCl, 5 mM EDTA, 5 mM EGTA, 0.5% NP-40, 40 mM β-glycerolphosphate, 0.5 µg/ml each Leupeptin, Pepstatin, Chymostatin, 50 mM NaF, 5 mM Na_3_VO_4_, with 1 µM microcystin) for 10–20 minutes on ice. Insoluble material was removed by centrifugation at 20 g for 10 minutes and supernatants were aliquoted and snap frozen in liquid nitrogen. Protein concentrations were determined using a Coomassie based method (Bio-Rad). Equal amounts of cellular protein (60 µg) were separated on 10% acrilamide gel and subsequently transferred on Hybond ECL nitrocellulose membrane (Amersham Biosciences). Total protein was visualized with Poinceau (Sigma). After blocking the membranes in TBS containing 5% BSA, 0.05% Tween 20, 0.02% NaN_3_ for 1 hour, primary antibodies were added in block solution for overnight incubation at 4°C. After five times five minute washes in TBS, 0.05% Tween 20, the appropriate HRP-conjugated secondary donkey antibodies (Amersham Biosciences) were added (dilution 1∶5000) for 30 minutes. After five washes (five minutes each) antibody binding was detected using ECL reagent (Amersham Biosciences). The sources and dilutions of the primary antibodies used for western blotting analysis are stated in the Supplementary Table S1.

### Immunohistochemistry

Immunohistochemistry was performed on formalin fixed tissue (Lkb1 immunohistochemistry was performed on methacarn fixed tissue). 3×1 cm gut fragments (7–10 cm away from the stomach) were flushed with water, bound in surgical tape and fixed in formalin or methacarn for a maximum of 14 hours at 4°C. All fixed samples were embedded in paraffin and sectioned to 5–6 µm on poly L-lysine slides for immunohistochemical analysis. Antigen retrieval was performed by boiling in citrate buffer (LabVision) for 10 minutes at 100°C. The endogenous peroxidase was blocked by EnVision blocking solution (DakoCytomation) for 5 minutes. The non-specific binding was blocked for 30 minutes with either 5% goat serum (DakoCytomation) for the primary rabbit polyclonal antibodies or 5% rabbit serum (DakoCytomation) for the primary mouse monoclonal antibodies and primary antibody incubation was performed at 4°C overnight. The detection was performed using secondary antibody horseradish peroxidase-labelled polymer and DAB reagent (DakoCytomation). The sources and dilutions of the primary antibodies used for immunohistochemical analysis are stated in the Supplementary Table S2.

## Supporting Information

Table S1 Antibodies used for western blot analysis.
(0.03 MB DOC)

Table S2 Antibodies used for immunohistochemical analysis.
(0.03 MB DOC)
