# Correction: Lkb1 Deficiency Alters Goblet and Paneth Cell Differentiation in the Small Intestine

**DOI:** 10.1371/annotation/7750701a-2069-4810-820b-b0a02b674a06

**Published:** 2009-02-05

**Authors:** Boris Y. Shorning, Joanna Zabkiewicz, Afshan McCarthy, Helen B. Pearson, Douglas J. Winton, Owen J. Sansom, Alan Ashworth, Alan R. Clarke

The last line of the Author Contributions is incorrect. It should read: Wrote the paper: BYS JZ AC.

