# Correction: Lkb1 Deficiency Alters Goblet and Paneth Cell Differentiation in the Small Intestine

**DOI:** 10.1371/journal.pone.0223041

**Published:** 2019-09-19

**Authors:** Boris Y. Shorning, Joanna Zabkiewicz, Afshan McCarthy, Helen B. Pearson, Douglas J. Winton, Owen J. Sansom, Alan Ashworth, Alan R. Clarke

After publication of this article [1], concerns were raised that in the Hes1 panel of Fig 3A, the background appears different in lane 2 than in other lanes.

The authors clarified that lanes were spliced out of the original blot image in preparing this Hes1 panel. The original blot is provided here as Supporting Information (S1 File). The authors apologize for not clearly noting the splicing lines in the published figure.

The authors further noted that the β-actin blot shown in Fig 3A is the loading control for the Hes5 experiment but not for the Hes1 experiment (although the same samples were loaded on both gels). Loading control data for the Hes1 blot are no longer available. The authors noted that the same “day 6” protein samples were used to load the gels but that the Hes5 and β-actin blots were done at a later time than the Hes1 experiment.

Fig 3C of this article examined levels of phosphorylated MARK and AMPK. Although a β-actin control blot was included the authors did not provide control blots showing total levels of MARK and AMPK.

The raw data are fully available for all the histology and immunostainings shown in the published article, but the original data underlying other western blot panels are no longer available.

In addition, the corresponding author listed on the published article [1] is deceased. Dr. Boris Shorning is now serving as the corresponding author, any queries should be directed to shorningB@cardiff.ac.uk.

## Supporting information

S1 File Original blot image underlying the Hes1 panel in Fig 3A. Lanes 2, 3, 6, 7 were used in the published figure.
(JPG)
