# Supplementary material for: Lkb1 Deficiency Alters Goblet and Paneth Cell Differentiation in the Small Intestine
Source: PLoS One. 2009 Jan 23;4(1):e4264. doi: 10.1371/journal.pone.0004264 (PMC2626247; doi:10.1371/journal.pone.0004264)
Supplement: Table S1 — Antibodies used for western blot analysis. (0.03 MB DOC) [file pone.0004264.s001.doc]

Supplementary Table S1

Antibodies used for western blot analysis

| Antigen | Dilution | Origin | Supplier |
| --- | --- | --- | --- |
| beta-actin | 1:5000 | mouse monoclonal | Sigma |
| Hes1 | 1:1000 | rabbit polyclonal | Chemicon |
| Hes5 | 1:2000 | rabbit polyclonal | Chemicon |
| Lkb1 | 1:1000 | mouse monoclonal | Upstate (clone5c10)) |
| phospho-AMPK (Thr 172) | 1:1000 | rabbit polyclonal | Cell Signaling Technology |
| phospho-MARK family (activation loop) | 1:1000 | rabbit polyclonal | Cell Signaling Technology |
