# Supplementary material for: Lkb1 Deficiency Alters Goblet and Paneth Cell Differentiation in the Small Intestine
Source: PLoS One. 2009 Jan 23;4(1):e4264. doi: 10.1371/journal.pone.0004264 (PMC2626247; doi:10.1371/journal.pone.0004264)
Supplement: Table S2 — Antibodies used for immunohistochemical analysis. (0.03 MB DOC) [file pone.0004264.s002.doc]

Supplementary Table S2

Antibodies used for immunohistochemical analysis

| Antigen | Dilution | Origin | Supplier |
| --- | --- | --- | --- |
| Beta-galactosidase | 1:500 | rabbit polyclonal | Abcam |
| Dll1 | 1:200 | rabbit polyclonal | Santa Cruz |
| Hes5 | 1:500 | rabbit polyclonal | Chemicon |
| Lkb1 | 1:200 | rabbit polyclonal, C-term | Abgent |
| Lysozyme | 1:200 | rabbit polyclonal | DAKO |
| Mark1 | 1:100 | rabbit polyclonal | Abcam |
| phospho-AMPK (Thr 172) | 1:100 | rabbit polyclonal | Cell Signaling Technology |
| phospho-mTOR | 1:100 | rabbit polyclonal | Cell Signalling Technology |
| Phospho-p70 S6 Kinase Thr421/Ser424 | 1:100 | rabbit polyclonal | Cell Signalling Technology |
| Phospho-S6 Ribosomal Protein Ser 240-244 | 1:100 | rabbit polyclonal | Cell Signalling Technology |
