# Supplementary figures and images for: Correction: Lkb1 Deficiency Alters Goblet and Paneth Cell Differentiation in the Small Intestine
Source: PLoS One. 2019 Sep 19;14(9):e0223041. doi: 10.1371/journal.pone.0223041 (PMC6752793; doi:10.1371/journal.pone.0223041)

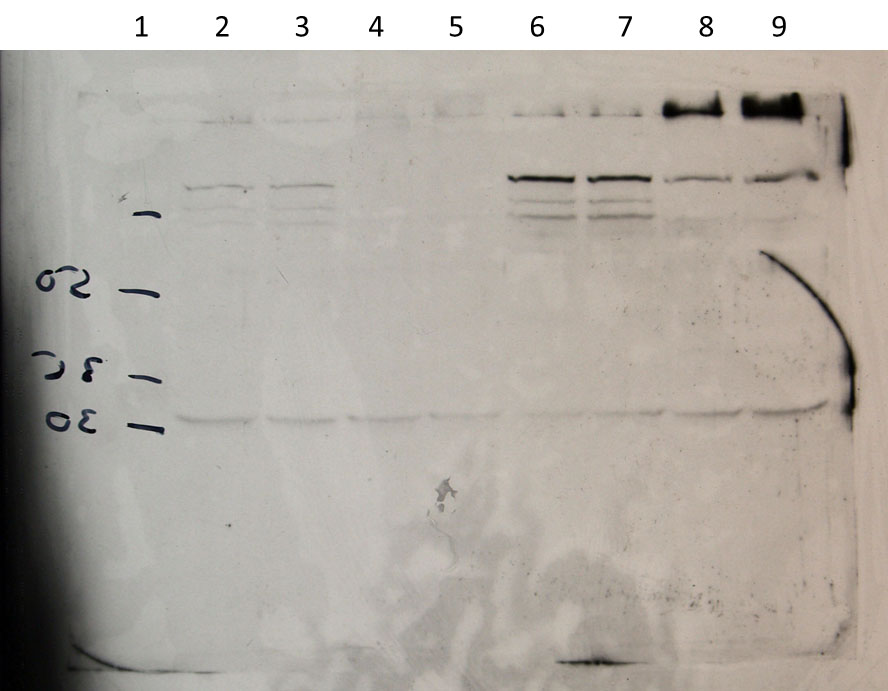

Supplement: S1 File — Lanes 2, 3, 6, 7 were used in the published figure. (JPG) [file pone.0223041.s001.jpg]
